# Notes and News

**Published:** 1893-11-11

**Authors:** 


### Notes and News.

COLLECTED AND COMMUNICATED.

MR. BURFORD RAWLINGS, Secretary of the National Hospital for the Paralysed and Epileptic, makes a seasonable appeal for hospital coal cellars. The coal famine has proved a very severe trial to the charities which, even in the time of plenty, have a hard enough fight to provide necessaries, and keep down expenses. The present crisis has proved the wisdom of the custom of one hospital secretary we know, who, profiting by a previous adverse experience, has always kept an untouched balance of one hundred tons of coal in his cellars. His forethought has not only been rewarded by the tiding over of a large portion of the present bad season, but he has further managed to alight on a find of coal which he has been able to procure at but a trifle above the usual cost.

THE Swansea Infirmary is in want of funds, a fact by no means uncommon with hospitals. The amount of its debt might not sound large to the ears of a metropolitan hospital secretary, but whilst it seems to us to be greater than it should be for a provincial institution, we cannot think the plan decided upon to increase its revenues at the last meeting is one likely to increase its popularity. Swansea has long held a Hospital Sunday collection. Now a Saturday is also to be introduced. Not only on the day in question are street collections to be made, but for some time prior it is arranged that children should carry on a house to house campaign. It is also proposed that ladies should sell flowers in the street at fancy prices for the good of the cause. It would seem, if all these methods are adopted, that energy is not lacking at Swansea. It will be well if the persistence displayed results in a substantial gain to an institution, which the evidence of a much increased Hospital Sunday collection last year proves to be gaining in popular favour. On the other hand, we would warn the hospital management that the real gain to the hospital of a street collection is very questionable whilst the irritation caused to the public, besieged as they are on such occasions, is very patent to all.

HOSPITAL Sunday at Birmingham does not show a brilliant result. The collection during the first few days amounted to £2,726 Last year it amounted to £2,802. Yet this year ten more congregations were contributing. The recipient charity, the Queen's Hospital, is badly in need of funds. In ten years the number of patients annually treated have increased by 13,088, and recent additions to the accommodation will add to this number. The subscription list to the hospital is actually smaller than in 1882.

THE report of the Howard Association for October, 1893, is full of the most interesting information. The field over which the Association casts an ameliorating influence is a large one, and the facts collected in the course of the Society's work make it possible for those who are willing and anxious to help their fellow creatures to decide in which direction they can best utilise their resources. The Report can be obtained at the offices of the Association, 5, Bishopsgate Street Without, and we recommend our readers to send for a copy.

THE Food and Cookery Exhibition at the Portman Rooms showed few new features. The absence of exhibits of cooking stoves of previous occasions made a space which was occupied by a display of army sick diets. There were specimens of the various provisions provided. Extras on sick diets included butter, arrowroot, milk, sago, sugar, rice, beef tea, egg flip, and custard pudding. Very special menus included lemonade and fish. The sample meals appeared excellent in quality, and certainly very ample in quantity. The son of Mars who acted as showman over this exhibit explained that tea was not given to sick people, as it acted injuriously on the coats of the stomach. Fearing that the little knowledge might prove a dangerous thing we refrained from endeavouring to extract further information from this quarter. The Army School of Cookery, which hails from Aldershot, proves that the right kind of attention is now being directed to culinary arrangements in the army. The men have better food at a reduction of expenditure. At the present time wholesome soups are made, and dripping and bones turned to account, which formerly were treated as so much waste material.

WE heartily congratulate the Medical, Sickness, Annuity, and Life Society, and Mr. Hart, on the success which is attending the association. Continual proofs are received of the appreciation in which the work is held by the medical profession. The sickness branch is of utmost benefit, and members of five years standing may now have their sick pay increased to £6 6s. per week. A good addition to the funds has been made during the year, the total assets now standing at £69,024. As a friendly society, however, the benefits offered to members were naturally limited, but with a business aptitude which is highly commendable, Mr. Hart has now succeeded in making arrangements with a leading Life Assurance Company under which members of the society can through it effect assurance on their lives, and secure deferred annuities on peculiarly favourable terms. This is a matter for great thankfulness that members of the medical profession through this society, and members of the nursing profession through the Royal National Pension Fund for Nurses, are now adequately safeguarded against the consequences of the risks which they are called upon to face in the battle they wage against sickness.

